# Association of Single-Nucleotide Polymorphisms in Interleukin Genes with Microbial Keratitis in a South Indian Population

**DOI:** 10.3390/pathogens11111387

**Published:** 2022-11-20

**Authors:** Nagaraju Konda, Subhabrata Chakrabarti, Prashant Garg, Mark D. P. Willcox

**Affiliations:** 1 School of Optometry and Vision Science, University of New South Wales, Sydney 2052, Australia; 2 Brien Holden Vision Institute, Sydney 2052, Australia; 3 School of Medical Sciences, University of Hyderabad, Hyderabad 500046, Telangana, India; 4 Brien Holden Eye Research Centre, L. V. Prasad Eye Institute, Hyderabad 500034, Telangana, India; 5 The Cornea Institute, L. V. Prasad Eye Institute, Hyderabad, Telangana 500034, India

**Keywords:** keratitis, genotype, SNP, interleukin

## Abstract

Background: To examine the relationship between single-nucleotide polymorphisms (SNPs) in interleukin (IL) genes and keratitis and its clinical manifestations. Methods: SNPs in *IL1B*, *IL6*, *CXCL8*, *IL10,* and *IL12B* were analysed. Differences in frequencies of alleles, genotypes and haplotypes between cases and controls as well as associations between SNPs and clinical variables were calculated by χ2 tests with odds ratios. Results: The minor homologous genotype in *IL1B* rs16944 (*p* = 0.036; odds ratio (OR) = 2.063, 95% confidence interval (CI): 1.048–4.061) and *CXCL8* rs4073 (*p* = 0.041; OR = 0.463, 95% CI: 0.224–0.956) and the heterologous genotypes in *IL6* rs1800795 (*p* = 0.046; OR = 0.563, 95% CI: 0.326–0.972) and *IL12B* rs2569254 (*p* = 0.0446; OR = 0.557, 95% CI: 0.314–0.989) or rs730691 (*p* = 0.0051; OR = 0.451, 95% CI: 0.260–0.784) were associated with keratitis. The minor genotype of rs16944 was associated with severe infection (*p* = 0.046). The heterologous genotype in rs2569254 was associated with hospital admission, photophobia, and mode of contact lens wear (*p* ≤ 0.041). The heterologous genotype in rs730691 was associated with blurred vision, discharge, anterior chamber reaction, and mode of wear (*p* ≤ 0.047). Conclusions: This study demonstrates that SNPs in *IL1B* and *CXCL8* are associated with risk of developing keratitis. The study also found relationships between SNPs and clinical measures of keratitis. The potential for ethnic differences in frequency of SNPs and their association with keratitis should be followed up using different populations.

## 1. Introduction

Microbial keratitis, infection of the cornea, occurs at a rate of 2.5–799 cases per 100,000 population/year. The estimated incidence in developed countries is 2.5–40.3 per 100,000 population/year [1,2,3,4,5,6,7]. However, the incidence in developing nations is higher at 113 per 100,000 in south India [8] and 799 per 100,000 in Nepal [7]. Contact lens wear is one of the significant risk factors for microbial keratitis [9,10]. Contact lens wear is also associated with the development of non-infectious keratitis, also called sterile keratitis or corneal infiltrative events [11]. Microbial keratitis is most commonly caused by bacteria [12], and corneal infiltrative events are associated with bacterial colonisation of contact lenses [13]. 

The immune response is known to play a pivotal role during microbial keratitis. A robust inflammatory response is needed at the beginning of the infection to control microbial numbers. However, a continued intense inflammatory response contributes to pathology associated with the disease [14,15,16]. Many studies have been conducted to outline the contribution of cytokines to the inflammatory response during microbial keratitis, especially in relation to infection caused by *Pseudomonas aeruginosa* in mouse models. For example, IL-1β [17,18,19,20], IL-1Ra [21], IL-4 [22,23], IL-6 [17,24,25], IL-8 (CXCL2) [26,27], IL-10 [28,29], IL-12 [30], IL-17 [31,32,33], IL-23 [34], IL-24 [35], IL-33 [36], and IL-36Ra [21] play roles in *P. aeruginosa* and other bacterial keratitis. IL-1β [37], IL-4 [38], IL-6 [39,40], IL-10 [41], IL-12 [38], IL-17 [42,43,44], IL-23 [39,40], and IL-33 [45] have roles in fungal keratitis. IL-1α [46], IL-2 [47], IL-6 [48,49], IL-10 [46], IL-12 [50], IL-17 [46,51], IL-18 [52], and IL-23 [53] have roles in viral keratitis and IL-17 [54] in *Acanthamoeba* keratitis. The interleukins IL-1α [55], IL-1β [55,56,57], IL-6 [57,58], IL-8 [57,59,60], and IL-17 [61] have been shown either at the mRNA or protein level to be produced during human microbial keratitis or resolution of disease. 

The contribution of interleukins to disease can also be examined by determining whether single-nucleotide polymorphisms (SNPs) within interleukin genes are associated with susceptibility to or severity of keratitis. The first study to examine whether SNPs in interleukins were associated with keratitis examined several SNPs in *IL10*. The *IL10* SNP rs6703630 allele A, which had been associated with a low IL-10 production, was more common in patients with corneal ulcers than in controls [62]. The *IL10* promotor SNP rs6693899 allele C, which had been associated with the production of high levels of IL-10, was associated with better clinical outcome of keratitis, whereas allele A of rs6693899 was associated with poor clinical outcomes [62]. A haplotype (i.e., a group of alleles that are inherited together) of IL-10, IL-10.1 (alleles CGAA of the SNPs rs 3021097, rs1800896, rs6693899, and rs6703630), was associated with worse clinical outcome, whereas the haplotype 10.2 (alleles CACG of the same SNPs) was associated with better clinical outcome of keratitis [62]. Subsequently, another group found that the minor alleles or genotypes of *IL10* SNPs rs1800871, rs1800896 or rs1800872 when analysed singly (haplotype analysis was not performed), were not associated with the severity of microbial keratitis, but SNP rs2397084 of *IL17F* showed a trend for increased severity of keratitis [63]. 

The minor *IL6* C allele of rs1800795 and T allele of rs1800797 were associated with a greater risk of microbial keratitis compared with sterile keratitis and controls combined, and the dominant haplotype GGC was associated with a lower risk of microbial keratitis [64]. When cases of keratitis were stratified on the basis of severity (sterile plus mild versus moderate plus severe), the people carrying the minor alleles in *IL6* rs1800795 or rs1800797 and people with the heterologous or homologous minor genotype in these SNPs were more likely to have a moderate/severe event, whereas people carrying the dominant haplotype in the *IL6* SNPs were less likely to have a severe event [64]. There was no association between the *IL6* SNP rs1800796 or *IL1B* SNP rs1143627 allele or genotype distributions and the type of condition or severity of the condition [64]. There was an association between a potentially deleterious missense SNP in the IL-6 signal transducer rs2228046 and keratitis in a small pilot study [65]. 

The dominant SNP rs3212227 in *IL12B* has been associated with a lower risk of having sterile keratitis compared with controls (with sterile keratitis being defined as no positive corneal culture or, where culture was not performed, a corneal infiltrate and overlying epithelial defect outside of the central 4 mm of the cornea, no uveitis, and no significant pain) [64]. A significant association was observed between recurrent Herpes simplex keratitis and SNPs of the *IL28B* genotype rs12979860 [66]. SNPs in *IL1B* (rs16944), *CXCL8* (rs2227307, rs2227543, and rs1126647), and *IL22* (rs1179251) have been associated with decreased risk of severe inflammatory complications during *Acanthamoeba* keratitis [67]. SNP rs9861402, near the gene encoding *CX3CR1* (the receptor for the chemokine fractaline), has been associated with keratitis in a human genome-wide study [68].

These previous studies examined mostly Caucasian populations. Therefore, the current study aimed to examine whether differences in SNPs in several interleukins that had been studied in humans or interleukins that had been shown to be associated with keratitis in animals were associated with keratitis in a south Indian population.

## 2. Materials and Methods

### 2.1. Study Participants 

The study was approved by the Human Research Ethics Committee, University of New South Wales, Sydney, and the Institutional Review Board at the L. V. Prasad Eye Institute (LVPEI), Hyderabad, India, and conformed to the tenets of the Declaration of Helsinki. All cases and controls were identified from the existing medical record database or the outpatient department of The Cornea Institute at LVPEI. All cases and controls had to be 18 years old or over, with no upper limit, and have no evidence of posterior segment abnormalities or systemic diseases, such as diabetes or hypertension. Cases had been diagnosed with microbial keratitis or a contact lens infiltrative event [69] All controls had no history of any form of keratitis. 

Sample size calculation indicated that 145 cases were required to determine an odds ratio (OR) of 2.34 for SNPs distributed at 20 percent based on a 1:1 ratio of controls to cases. The Type I error probability associated with testing the null hypothesis was set at 0.05, with a power of the study at 80% (PS Power and Sample Size Calculation Program Version 3) [70].

After obtaining informed consent, 145 cases and 189 controls were enrolled. Eighty-one cases had been diagnosed with microbial keratitis of bacterial origin (70% confirmed by culture; 32% caused by *P. aeruginosa,* 20% by *Streptococcus pneumoniae,* or classified based upon clinical presentation and response to antibiotic therapy), and 64 cases had been diagnosed as having had a corneal infiltrative event during contact lens wear (63% infiltrative keratitis (IK), 25% contact-lens-induced peripheral ulcer (CLPU), and 12% contact-lens-induced red eye (CLARE)) [69]. The diagnosis for contact lens induced inflammatory events was based on previously published criteria [11].

Clinical records of all cases and controls were examined. Data were collected for the following variables: mode of contact lens wear (daily wear only or any extended wear), admission to hospital to treat the keratitis (yes, no), presentation at the emergency clinic (yes, no), duration of the event until resolution (days), best corrected visual acuity (VA) at presentation (converted to decimal), best corrected VA at the resolution of the event (converted to decimal), vision loss (best corrected VA at resolution minus best corrected VA at presentation), VA loss (yes, no), watering, pain, irritation, conjunctival redness, lid edema, conjunctival edema, photophobia, blurred vision, discharge (all preceding nine items: yes, no), congestion (0–4), anterior chamber reaction (0–4), epithelial defect width (mm), infiltrate width (mm), and contact lens wear duration (months). An overall disease severity score was calculated by summing scores of all variables.

### 2.2. SNP Selection and Analysis

Aliquots of 2–3 mL venous blood were collected in Vacuette EDTA tubes (Greiner bio-one, Frickenhausen, Germany) and stored at −20 °C. Genomic DNA was extracted using MagNA Pure LC (Roche Applied Science, Mannheim, Germany) following the manufacturer’s instructions. Quant-iT™ PicoGreen^®^ dsDNA fluorescent nucleic acid stain (Invitrogen, Ltd., Paisley, UK) was used to quantify the dsDNA.

The interleukins of interest were selected on the basis of their association with keratitis in either human studies (IL-1β [55,56,57,64], IL-6 [57,58,64], IL-8 [57,59,60,68], or IL-10 [62]) or in mouse models of bacterial keratitis (IL-1β [17,18,19,20], IL-6 [17,24,25], IL-8 (CXCL8) [26,27], IL-10 [28,29], or IL-12 [30]). The candidate SNPs (Supplementary Table S1) in the genes for these interleukins were selected on the basis of an associated allele frequency of ≥ 0.5, with reference to online databases (db SNP (http://www.ncbi.nlm.nih.gov/projects/SNP; accessed on 11 April 2013), Hap map data base (http://hapmap.ncbi.nlm.nih.gov; accessed on 11 April 2013), and Indian genome variation database http://www.igvdb.res.in; accessed on 11 June 2020)). Flanking variants to the known SNPs at a distance of 500 bp at the 5′ and 3′ ends were also included. A total of 40 SNPs were selected on the basis of the above criteria and synthesized for custom genotyping (Illumina Inc., San Diego, CA, USA).

Screening for SNPs in the participants’ DNA used Illumina’s GoldenGate^®^ assay, following the manufacturer’s instructions. The SNPs highlighted in grey cells in Supplementary Table S1 were either monomorphic or had improper data clustering (*IL12B* rs3213096 only) in the GoldenGate^®^ assay and were excluded from further analysis. GenomeStudio™ software (Illumina Inc., Version 20.10) was used to extract the data generated from the GoldenGate^®^ assay. The GenTrain score, a value between 0 and 1, indicated the reliability of each genotype with a score of 0 indicating the location of the genotype was furthest from the cluster and a score of 1 indicating its being closest to the centre of the cluster. GenTrain score is determined on the basis of the angle, dispersion, overlap, and intensity of each SNP. A cut-off of 0.15 was set as a GenTrain score in the current study. Call rate is the fraction of genotypes passed from the total of successfully genotyped samples. These values vary between 0 and 1. Replicate samples from each batch were compared and manually edited, where necessary, with the help of the SNP graph and clustering of the SNP genotype. The average GenTrain and call rates for the SNPs used in the study are listed in Supplementary Table S1. 

IL-10 SNPs rs6693899 and rs6703630 were not included in the GoldenGate assay and so were analysed using PCR. Their sequences were obtained from the dbSNP database (http://www.ncbi.nlm.nih.gov/SNP/; accessed on 11 April 2013) and primers were designed using primer 3 software (version 0.4.0; http://frodo.wi.mit.edu/primer3/; accessed on 11 April 2013) and obtained from Eurofins (Bengaluru, Karnataka, India). Primer-BLAST (Basic Local Alignment Search Tool) was used to confirm the absence of non-specific binding. IL-10 primers were 1F-ACTCAGGGATGCAGGCAGCCT and 1R- TTGCACCAGGGAACTTGCCCA for rs6693899, and 1F- ACTCAGGGATGCAGGCAGCCT and 1R- TTGCACCAGGGAACTTGCCCA for rs6703630. Sequencing PCR used BigDye^®^ terminator cycle sequencing kit (Thermofisher, Bengaluru, Karnataka, India). Sequencing was performed in a thermal cycler (Veriti™, 9700, 1761 Applied Biosystems, Inc., Foster City, CA, USA) under the following conditions: initial denaturation at 96 ^o^C for 2 min, 30 cycles of denaturation at 96 ^o^C for 10 s, annealing at 50 ^o^C for 6 s, and extension at 60 ^o^C for 4 min, with a final hold at 4 ^o^C for 10 min. Samples were processed for sequencing using a 16-capillary sequencer (3130 × 1 Genetic Analyser, Applied Biosystems. Inc. Waltham, Massachusetts, USA). Electropherograms were analysed using the online software Chromas (Version 2.33), and the sequence was compared to wild-type gene sequence obtained from the NCBI database (http://www.ncbi.nlm.nih.gov/; accessed on 11 April 2013), and differences were noted.

### 2.3. Statistical Analysis

Allele and genotype frequencies were counted. Hardy–Weinberg equation (HWE) was estimated for the normal controls, and linkage disequilibrium (LD) between the variants was calculated by the LD plot function of Haploview software (version 4.2; http://www.broadinstitute.org/; accessed on 11 April 2013). The cases were classified as any type of keratitis (inflammation and infection), microbial keratitis (corneal infection; MK) only, or sterile keratitis (CLARE, CLPU, and IK; SK) only. The differences in frequencies in cases and controls were calculated by Chi-square (χ2). *p*-values were corrected for multiple SNP analyses using the permutation test in plink (https://www.cog-genomics.org/plink/; accessed on 16 June 2020) on the basis of the maxT procedure, using 100,000 permutations. Haplotype frequencies were calculated using the SHEsis software [71]. Haplotypes distributed at frequencies of > 3% in the dataset were considered, and the odds ratio was calculated to analyse the association for all cases and controls. In the haplotype analysis, control of multiple analyses used: corrected *p* = 1-(1-Fisher’s *p*-value)^N^, where N= number of comparisons.

For comparisons of clinical differences between cases and controls within different genes (SNPs) or combinations of SNPs within genes, χ2 test with Yates correction for multiple comparisons was carried out; odds ratios were calculated for the significantly different variables.

## 3. Results

### 3.1. Subject Demographics

This study used the same subjects as a previous study that examined their DNA for SNPs in several Toll-like receptors [69]. In brief, all were of Indian descent, and most were from Hyderabad, Telangana, India. The average age of all cases (32.33 ± 13.52 years) was not significantly different from controls (30.10 ± 11.07; *p* = 0.104). The majority of cases (61%) and controls (68%) were male.

### 3.2. Analysis of Candidate Genes

The monomorphic SNPs in this population (rs78226748, rs11544633, rs34012176, rs2227532, rs3213119, rs74644143, rs79446920, and rs10045130) and rs3213096, which had improper data clustering in the GoldenGate^®^ assay, were not analysed further. The linkage disequilibrium (D’) between adjacent SNPs in each of the candidate genes was calculated (Supplementary Table S2). Most SNPs within a gene were in high linkage disequilibrium. There were no departures from HWE for any of the polymorphic variants (*p* > 0.05) in the control population. Supplementary Figure S1 shows the position of the SNPs within each interleukin gene.

The significant allele and genotype differences between each case compared with controls is shown in Table 1 (with the full data set shown in Supplementary Table S3). There were no significant differences in alleles. When all cases were combined and compared with controls, there were significant differences in genotypes for *IL1B* rs16944 (*p* = 0.036), with the homozygous minor genotype being more likely in cases (OR = 2.063); *IL6* rs1800795 (*p* = 0.046), with the heterologous minor genotype (OR = 0.563); and *CXCL8* rs4073 (*p* = 0.041), with the heterologous minor genotype (OR = 0.463) being less likely to occur in cases. For *IL12B*, the rs2569254 SNP (*p* = 0.0446), with the heterologous minor genotype (OR = 0.557), and the rs730691 SNP (*p* = 0.005), with the heterologous minor genotype, were less likely to occur in cases (OR = 0.451; Table 1). There were also three SNPs that showed a trend (*p* < 0.1; Table 1) for differences between cases and controls, with *IL1B* rs1143627 homologous minor genotype (OR = 1.925; *p* = 0.0639); *CXCL8* rs2227307, with the homologous minor genotype (OR = 0.518; *p* = 0.0929); and *CXCL8* rs2227543, with the homologous genotype (OR = 0.492; *p* = 0.0879), being less likely to occur in cases.

Other analyses were performed after separating microbial keratitis (infection cases) and sterile keratitis and then comparing with the control population or each other. Table 2 shows the significantly different data, with the full data set in Supplementary Tables S3–S6. The analysis of MK only versus controls showed very similar differences as with the full case vs. control analysis, with the exception that the SNP in *IL6* was this time rs10499563, with its homologous minor genotype showing a trend to occur more frequently in cases (OR = 2.95; *p* = 0.093). When SK only cases were compared with controls, there was only one significant difference in SNPs (Table 2), and that was for the *IL6* SNP rs1554606, which had a trend for being found more frequently in cases than controls (OR = 2.74; *p* = 0.09; Table 2). There were no significant differences (or trends) in the frequency of any SNP in any interleukin between MK and SK cases.

The data were further analysed to assess whether the associated variants within an IL gene co-occurred more in cases than controls. Co-occurrence of the homozygous minor alleles (OR = 18.58; 95% CI: 6.54–52.79; *p* < 0.00001) or genotypes (OR = 18.58; 95% CI: 4.24–81.36; *p* < 0.00001) in the two associated SNPs of *IL1B* (rs16944 and rs1143627) were associated with increased risk of keratitis. The co-existence of the heterologous minor alleles (OR = 0.32, 95% CI: 0.14–0.71; *p* = 0.0038) or genotypes (OR = 0.24; 95% CI: 0.1–0.58; *p* = 0.0018) of *IL12B* were associated with a significant reduction in the risk of keratitis. In the case of *CXCL8*, the controls did not exhibit homozygosity across any of the three SNPs, and so, this analysis could not be performed. This analysis was not performed for MK and SK cases, as the number of these cases was too small.

The overall genetic interactions across all the IL SNPs indicated that the random presence of more than three homozygous mutant variants across any of the IL-associated genes increased the disease risk of all types of keratitis by more than sixfold (OR = 6.11, 95% CI: 1.71–21.85; *p* = 0.012), but the presence of less than three homozygous mutant variants across the spectrum of IL genes in an individual conferred protection (OR = 0.53, 95% CI: 0.33–0.85; *p* = 0.0025). This analysis was not performed for MK and SK cases, as these did not indicate strong associations to any of these variants.

The data were also analysed for differences in haplotypes which occurred in any population at a frequency of >3% for each interleukin. There were no significant differences in haplotypes between any cases and controls or between SK and controls in any interleukin with any combination of SNPs within any of the individual genes. The only differences were found when MK was compared with controls or SK cases (Table 3). Two haplotypes of *IL10* occurred more frequently in MK cases than in controls, and one of the same haplotypes occurred more frequently in MK compared with SK cases (Table 3).

### 3.3. Association with Clinical Variables

There were various associations between possession of SNPs in cases and clinical variables (Table 4). Cases of keratitis with the minor genotype in SNP rs16944 (*p* = 0.046; OR = 4.02) of *IL1B* were more likely to have a higher score for severity of the disease than cases that possessed the dominant genotype. For both the *IL12B* SNPs (rs2569254 and rs730691), the cases with the minor heterologous genotypes were more likely to wear contact lenses on an extended wear than daily wear basis (*p* = 0.041, OR = 4.74; *p* = 0.009, OR = 6.37, respectively). In addition, cases with the minor genotype in rs2569254 were more likely to report photophobia during the keratitis (*p* = 0.015, OR = 5.78). The other factors associated with possession of the minor heterologous genotype were hospital admission for rs2569254 (*p* = 0.012, OR = 5.29) and for rs730691, more likely to have more severe blurred vision (*p* = 0.047, OR = 3.04) and anterior chamber reaction (*p* = 0.025, OR = 5.68), but less likely to have discharge (*p* = 0.016, OR = 0.19).

## 4. Discussion

This study has shown several significant differences in the carriage of SNPs in interleukin genes between people who had keratitis and controls. Two SNPs in *IL1B*, rs1143627 and rs16944, occurred more commonly in all cases of keratitis, and even though the statistical power would have been reduced due to the decrease in sample size, these differences also occurred when only cases of microbial keratitis were compared with controls. Certain SNPs in *IL6*, *CXCL8*, and *IL12B* occurred less frequently in all keratitis cases than in controls. Even though the statistical power would have been reduced due to a decrease in the sample size, one SNP in *IL6* occurred significantly more frequently in cases of sterile keratitis than in controls. Whilst no single SNP in the *IL10* gene was different between cases and controls, two haplotypes of *IL10* occurred more frequently in cases of microbial keratitis than controls, and one of these also occurred more frequently in cases of microbial keratitis compared with sterile keratitis. Cases that possessed the minor genotypes were often more likely to be associated with various clinical and other responses during the disease than cases that possessed the dominant genotypes.

IL-1β has been shown to be a key orchestrator of the inflammatory response during bacterial keratitis. IL-1β can increase the production of matrix metalloproteases during *P. aeruginosa* keratitis, and increases in these proteases were associated with worse clinical outcomes [20]. Preventing the production of IL-1β during *P. aeruginosa* keratitis by silencing the gene for ﻿the inflammasome NLRC4 [18], inhibiting caspase-1 production (either using gene knockout mice or antibodies) [19,72], or administering IL-1Ra (a natural inhibitor of IL-1β) [21] reduces the clinical pathology of keratitis. The two SNPs, rs1143627 and rs16944, in *IL1B* were in high linkage disequilibrium in the current study, suggesting they are almost always inherited together and were more frequent in the keratitis and MK groups. People with a haplotype of these two SNPs containing the minor alleles have 2–3 times increased production of IL-1β in blood when stimulated with lipopolysaccharide [73], and a significant association has been found between the presence of the A polymorphic allele (genotypes GA and AA) in rs16944 and higher IL-1β mRNA expression [74]. It may be that this also increases the local production of IL-1β during keratitis. This needs to be tested in follow-up experiments. The finding that cases with the minor genotype in rs16944 were more likely to be classified as having severe disease than cases without the minor genotype (*p* = 0.046; OR = 4.02) supports a possible role in increased production of IL-1 leading to increased severity of the disease.

Interestingly, these two SNPs of *IL1B* have also been associated with daytime napping in adults living with HIV [75]. With the well-known increase in the risk of keratitis when people sleep in contact lenses [76], it would be interesting to determine whether the people with these SNPs were also more likely to nap during contact lens wear. However, it was noted that the cases that possessed these two SNPs were not more likely to report flexible (i.e., occasional use during the night) or true extended wear (i.e., sleeping in lenses) than cases that did not possess these SNPs. Reduced frequency of the minor allele in rs16944 has been linked with protection from severe inflammatory complications in *Acanthamoeba* keratitis [59], whereas in the current study, there was no allelic association with any form of keratitis nor when MK (a more severe form of keratitis) was compared with SK. In a previous study, the *IL1B* SNP rs1143627 was not associated with MK [64] This difference to the current study may be due to differences in the frequency of the minor allele or homologous genotype between the two studies in the control populations: 29.2% and 5.9% versus 38.6% and 10.6%, respectively.

IL-8 (CXCL8) and the mouse equivalents, MIP-2 and KC, have been shown to be key chemokines that function during keratitis to attract polymorphonuclear leucocytes to the cornea and tear film [27,57,59,60,77]. These studies had shown that prolonged production of IL-8 can exacerbate the pathology during microbial keratitis. In the current study, the TT genotypes of both rs4073 and rs2227543 were less frequent in all cases and trended for a decreased frequency in MK cases versus controls for rs4073. A study examining associations between interleukin SNPs and severity of inflammatory complications during *Acanthamoeba* keratitis found that the TT genotype of rs2227543 was less frequent in severe disease [59]. In a study from Korea, the TT genotype for SNP rs4073 was associated with higher production of IL-8 in serum [78], and another study found the TT genotype of SNP rs2227543 was associated with an increased amount of IL-8 for Pakistani subjects [79]. However, in another study, there was no difference in IL-8 production with people of Greek origin with different genotypes of rs4073 [80]. It is not known how these SNPs affect the production of IL-8 in an Indian population, but the reduced frequency in cases might indicate that they have a more likely chance of reduced production of IL-8, which helps to resolve the disease. The study on *Acanthamoeba* keratitis did not find associations between any of these SNPs and the amount of IL-8 in tears [59]. As the timing of IL-8 production is critical for clinical outcomes of keratitis [14], it would be interesting to determine whether people with these SNPs have a changed timing for IL-8 production in ocular tissues or tears. Interestingly, the frequency of these SNPs in SK cases was not different from controls or from MK cases, with the frequency in SK cases (9.4% and 7.8% for both SNPs, respectively) being between those of MK cases (8.6% and 6.2%, respectively) and controls (18.5% and 13.8%, respectively). Future experiments should confirm any changes in the production of IL-8 in ocular surface cells or white blood cells that appear in the corneas during keratitis in people with these SNPs. The other *IL8* SNP, rs2227307, which was associated with protection from all types of keratitis and microbial keratitis, has not been associated with changes to serum IL-8 levels in a predominantly non-Hispanic white population [81]. 

A previous study showed that people who carry the *IL12B* homozygous dominant genotype in SNP rs3212227 were more likely to have had sterile keratitis during contact lens wear than a control population [64]. This finding was not replicated in the current study for SK, MK, or all cases versus the control population. This difference may be partly due to the different frequencies of the genotypes in the control populations between the two studies. In the previous study [64], the dominant TT genotype was present in 62.7% of the control population, whereas in the current study with the south Indian population, it was present in only 36.5% of the control population. The current study found that the presence of heterologous minor genotypes in SNPs rs2569254 or rs730691 was protective for all keratitis cases and MK cases only, but there was no association for SK only versus controls. These two SNPs have not been previously examined for association with keratitis. This association with the minor heterologous genotypes in these *IL12B* SNPs (and the SNP in rs1800795 in *IL6*) may be due to purifying selection against homologous deleterious recessive alleles, or heterologous alleles conferring optimal intermediate levels of expression, or that the heterologous state prevents the overshooting of the optimal gene expression levels. There was an allelic and genotypic protection with the minor allele of rs2569254 for allergic rhinitis in a Han Chinese population [82]. A study examining *IL12B* SNP rs12979860 showed an association with recurrent Herpes simplex keratitis [66], but another study examining *IL1B* SNPs rs3212227, rs10045431, and rs6887695 found no association with severity of inflammation during *Acanthamoeba* keratitis [59]. There are currently no reports linking rs730691 to other inflammatory conditions or infections. 

Having the heterologous genotypes was protective of keratitis, but if cases with the heterologous genotypes had keratitis, they were more likely to show an increase in some of the signs associated with the disease (blurred vision, anterior chamber reaction (rs730691), or photophobia (rs2569254)). The finding that cases with the minor genotypes in both rs2569254 and rs730691 were more likely to use contact lenses for extended or flexible wear (ORs = 4.74 and 6.37, respectively) is of interest. It seems unlikely that the minor genotype would directly cause a person to choose to use contact lenses on an extended or flexible wear basis. However, as extended or flexible wear in this population has been associated with the risk of having MK [83], this may have affected the differences seen with other clinical variables, such as photophobia, blurred vision, and anterior chamber reaction, all of which are commonly seen with MK [11,83].

A previous study has linked the minor alleles of the *IL6* SNPs rs1800795 or rs1800797 with a greater risk of MK compared with SK or controls [64], but these and rs1800796 were not associated with severity of inflammation during *Acanthamoeba* keratitis [59]. The current study did not replicate the previous associations but instead found that the heterologous SNP rs1800795 was associated with protection from MK compared with controls, the homozygous minor genotype in rs10499563 was associated with an increased risk of MK compared with controls, and the homologous minor genotype in rs1554606 was associated with an increased risk of SK compared with controls. This implies a possible contribution to ethnicity in the role of IL-6 in keratitis, or a different role of IL-6 in different forms of keratitis. Certainly, the populations had a different mix of allele and genotype frequencies for rs1800795, with the current study having the minor allele in 14.8% of the control population compared with 41.4% in the previous study [64] and the current study having the minor homologous genotype in 1.6% of the control population compared with 18.4% in the previous study [64]. Similarly, for rs1800797, the minor allele was present in 14.3% of the control population in the current study compared with 40.5% in the previous study, and the minor homologous genotype was present in 1.6% of the control population in the current study but 18.4% in the previous study [64].

The rs1800795 minor homologous genotype has been associated with a lower concentration of IL-6 in plasma, whereas the heterologous genotype has plasma levels similar to the homologous dominant genotype in an Italian population [84]. However, in an Indian population from Punjab, rs1800795 was associated with a lower level of circulating IL-6 in a dose-dependent manner, with the homologous minor genotype having lower levels than the heterologous genotype [85]. Furthermore, in a study from Pakistan, the homologous and heterologous minor genotypes also had lower levels of IL-6 than the dominant genotype [79]. Rs1800797 was not associated with a reduction in circulating IL-6 levels in the Punjabi population or Pakistani study, which is perhaps why this SNP was not associated with all keratitis, MK, or SK in the current study [79,85]. For MK only, the homologous SNP in rs10499563 showed a trend of association with disease. This SNP has also been associated with a reduction in circulating IL-6 in a Punjabi population [85]. If the circulating concentration of IL-6 is reduced by the minor alleles or genotypes in rs1800795 and rs10499563 in the current population, this appears to be not directly associated with the risk of keratitis, as the SNPs were associated with decreased and increased risk of MK, respectively. Perhaps the effect of the SNPs on the ocular surface concentration of IL-6 is different from their effects in the blood. This should be examined in future studies. The SNP rs1554606, which was associated with increased risk of SK, has also been associated with patients with HIV napping during the day. Again, it would be interesting to determine whether the people with this SNP were more likely to nap or sleep.

The current study was able to replicate the lack of association of the IL-10 SNPs rs1800872, rs1800896, and rs6703630 with risk of keratitis or severe keratitis [62,64]. In a previous study, the rs6693899 allele C (G in the current labelling), that had been associated with the production of high levels of IL-10, was associated with better clinical outcome of keratitis, whereas the allele A (T in current labelling) of the rs6693899 promoter SNP was associated with poor clinical outcome [62]. This was not replicated in the current study. Again, this may be due to the differences in allele and genome frequencies between the two populations. In the (presumably) predominantly Caucasian population of the previous study, the frequency of the minor allele was 40%, and the homozygous genotype was 16% [62], whereas in the current study of a south Indian population, these frequencies were 16.4% and 3.7%, respectively. The current study did demonstrate strong associations for two *IL10* haplotypes, AATTTATGTC and AGCGTCCAGC, with MK compared with controls and AGCGTCCAGC with MK compared with SK. The effect of these *IL10* haplotypes on IL-10 production is not known and should be studied. The association with a haplotype, but not a single allele or genotype, may be observed if there are associated SNPs in between the ones chosen for study. Since only a few chosen SNPs were analysed, there is a possibility of additional SNPs within this associated haplotype that may be associated with keratitis.

## 5. Conclusions

In conclusion, the current study reinforces the importance of IL-1β, IL-6, IL-8, IL-10, and IL-12B in bacterial keratitis that had previously been shown in animal studies [17,18,19,20,24,25,26,27,28,29,30] and in human SNP analyses [62,63,64,86]. The current study demonstrated that SNPs within *IL1B* and *CXCL8* are associated with risk of developing all forms of keratitis or (at least a trend in) microbial keratitis (frank bacterial corneal infection). The current study did not confirm the association of the homozygous dominant genotype in SNP rs3212227 of *IL12B* with sterile keratitis but did find that associations of two other SNPs in *IL12B*, SNPs rs2569254 or rs730691, were protective for all cases of keratitis and microbial keratitis. In addition, the current study did not confirm a previous finding that people with the *IL6* SNPs rs1800795 or rs1800797 were at greater risk of MK compared with SK or controls, but did find associations of other *IL6* SNPs (rs1800795, rs10499563, and rs1554606) with risk of any keratitis, microbial keratitis or sterile keratitis. The current study confirmed the lack of association of the SNP rs1800872, rs1800896 and rs6703630 in *IL10* with risk of keratitis or severe keratitis, and found two novel haplotypes of *IL10* were associated with risk of MK compared with controls. Future studies are needed to confirm the associations found in the current study. The potential for ethnic differences being associated with some of the associations with SNPs should be followed up using different populations. Furthermore, the frequency of some SNPs in the population were below the 20 percent used in the sample size calculations. The frequencies of all SNPs examined in the current study have been given in Tables and Supplementary Tables for use in future studies to generate appropriate sample sizes. 

## Figures and Tables

**Table 1:** Significant allele and genotype frequencies in cases compared with controls.

Gene	rs ID	Allele/Genotype	Frequency (%)		χ^2^ Value	Corrected *p*-Value	OR (95% CI)
			Cases (N = 145)	Controls (N = 189)			
*IL1B*	rs16944	A	58.6	64			Reference
		G	41.4	36	1.343	0.3107	1.26 (0.81, 1.96)
		AA	37.2	38.6			Reference
		AG	42.8	51.3	0.364	0.5325	0.864 (0.538, 1.389)
		**GG**	**20**	**10.1**	**4.397**	**0.036**	**2.063 (1.048, 4.061)**
	rs1143627	G	59.3	64.0			Reference
		A	40.7	36.0	1.392	0.4565	0.82 (0.52,1.28)
		GG	37.9	38.6			Reference
		GA	42.1	50.8	0.494	0.4835	0.843 (0.525, 1.356)
		***AA***	***20.0***	***10.6***	***3.683***	***0.069***	***1.925 (0.986, 3.756)***
*IL6*	rs1800795	G	88.3	85.2			Reference
		C	11.7	14.8	0.67	0.4136	0.76 (0.40, 1.46)
		GG	80	72			Reference
		**GC**	**16.6**	**26.5**	**4.257**	**0.046**	**0.563 (0.326, 0.972)**
		CC	3.4	1.6	0.817	0.4535	1.954 (0.457, 8.352)
*CXCL8 (IL8)*	rs4073	A	64.8	59.3			Reference
		T	35.2	40.7	0.605	0.3147	0.79 (0.50, 1.23)
		AA	39.3	37.6			Reference
		AT	51.7	43.9	0.245	0.6144	1.126 (0.705, 1.797)
		**TT**	**9**	**18.5**	**4.333**	**0.041**	**0.463 (0.224, 0.956)**
	rs2227307	T	66.9	64.0			Reference
		G	33.1	36.0	0.102	0.5794	0.88 (0.56, 1.39)
		TT	41.4	43.4			Reference
		TG	51.0	41.3	1.222	0.2777	1.297 (0.818, 2.055)
		***GG***	***7.6***	***15.3***	***2.799***	***0.0929***	***0.518 (0.240, 1.119)***
	rs2227543	C	70.3	66.1			Reference
		T	29.7	33.9	0.046	0.4156	1.21 (0.76, 1.94)
		CC	46.9	46.0			Reference
		CT	46.2	40.2	0.267	0.6134	1.128 (0.714, 1.781)
		***TT***	***6.9***	***13.8***	***3.054***	***0.0879***	***0.492 (0.222, 1.090)***
*IL12B*	rs2569254	C	90	85.71			Reference
		T	10	14.29	1.634	0.204	0.641 (0.323, 1.272)
		CC	82.76	74.07			Reference
		**CT**	**14.48**	**23.28**	**3.995**	**0.0446**	**0.557 (0.314, 0.989)**
		TT	2.76	2.65	0.01	0.8092	0.933 (0.245, 3.554)
	rs730691	T	49.7	53.4			Reference
		C	50.3	46.6	0.471	0.493	1.164 (0.755, 1.795)
		TT	28.3	25.4			Reference
		**TC**	**42.8**	**57.1**	**7.973**	**0.005**	**0.451 (0.260, 0.784)**
		CC	29	17.5	1.601	0.2128	0.671 (0.362, 1.245)

Data in bold type indicate significant differences (*p* < 0.05) between cases and controls. Data in bold and italics indicate trend for differences (*p* < 0.1) between cases and controls. rs id—Reference SNP cluster identification; OR—Odds ratio; and CI = confidence interval.

**Table 2:** Significant differences in alleles or genotypes of interleukin genes between cases of microbial keratitis (confirmed infections) or sterile keratitis and controls.

Gene	rs ID	Allele/Genotype	Frequency (%)		χ^2^ Value	Corrected *p*-Value	OR (95% CI)
			MK Only Cases (N = 81)	Controls (N = 189)			
*IL1B*	rs16944	A	58	64			Reference
		G	42	36	0.77	0.408	1.32 (0.77, 2.24)
		AA	38.3	38.6			Reference
		AG	39.5	51.3	0.62	0.380	0.76 (0.43, 1.36)
		**GG**	**22.2**	**10.1**	**3.34**	**0.036**	**2.20 (1.02, 4.75)**
	rs1143627	G	58.6	64.0			Reference
		A	41.4	36.0	0.36	0.486	1.22 (0.72, 2.09)
		GG	39.5	38.6			Reference
		GA	38.3	50.8	0.78	0.318	0.74 (0.41, 1.32)
		***AA***	***22.2***	***10.6***	***2.80***	***0.069***	***2.05 (0.96, 4.39)***
*IL6*	rs10499563	T	81.5	81.5			Reference
		C	18.5	18.5	0.03	1.000	1.00 (0.51, 1.95)
		TT	67.9	64.6			Reference
		TC	27.2	33.9	0.60	0.350	0.76 (0.43, 1.36)
		***CC***	***4.9***	***1.6***	***2.10***	***0.093***	***2.95 (0.64, 13.66)***
*CXCL2 (IL8)*	rs4073	A	66.7	59.3			Reference
		T	33.3	40.7	1.02	0.397	0.73 (0.42, 1.26)
		AA	40.7	37.6			Reference
		AT	50.6	43.9	0.02	0.811	1.00 (0.56, 1.75)
		***TT***	***8.6***	***18.5***	***2.70***	***0.062***	***0.43 (0.17, 1.07)***
	rs2227307	T	67.9	64			Reference
		G	32.1	36	0.22	0.584	0.84 (0.48, 1.46)
		TT	42.0	43.4			Reference
		TG	51.9	41.3	0.63	0.346	1.3 (0.75, 2.25)
		***GG***	***6.2***	***15.3***	***2.21***	***0.086***	***0.42 (0.15, 1.17)***
*IL12B*	rs2569254	C	91.4	85.7			Reference
		T	8.6	14.3	1.17	0.189	0.57 (0.24, 1.36)
		CC	85.2	74.1			Reference
		**CT**	**12.3**	**23.3**	**3.63**	**0.045**	**0.46 (0.22, 0.97)**
		TT	2.5	2.7	0.03	0.600	0.81 (0.15, 4.29)
	rs730691	T	53.1	53.4			Reference
		C	46.9	46.6	0.01	0.999	1.01 (0.6, 1.71)
		TT	32.1	25.4			Reference
		**TC**	**40.7**	**57.1**	**2.79**	**0.007**	**0.56 (0.31, 1.05)**
		CC	27.2	17.5	0.15	0.137	1.23 (0.6, 2.53)
			SK only cases (N = 65)	Controls (N = 189)			
*IL6*	rs1554606	G	82.8	82			Reference
		T	17.2	18	0	1.000	0.95 (0.45, 2.0)
		GG	71.9	66.7			Reference
		GT	20.3	30.7	1.51	0.164	0.61 (0.31, 1.22)
		***TT***	***7.8***	***2.6***	***1.51***	***0.090***	***2.74 (0.76, 9.9)***

Data in bold type indicate significant differences (*p* < 0.05) between cases and controls. Data in bold and italic type indicate trends for differences (*p* < 0.1) between cases and controls. rs id—Reference SNP cluster identification; OR—Odds ratio; and CI = confidence interval

**Table 3:** Significant differences in haplotypes between MK cases and controls or SK cases.

*IL10* Haplotype	Case (Frequency %)	Control (Frequency %)	χ^2^ Value	Corrected *p*-Value	Odds Ratio (95% CI)
**MK vs. controls**					
TATATTTCTC	7.11 (4.4)	1.92 (0.5)	12.029	0.000527	10.216 (2.045–51.021)
TGCGTGCTGC	14.13 (8.7)	13.91 (3.7)	7.711	0.005502	2.862 (1.325–6.183)
**MK vs. SK**					
TGCGTGCTGC	14.13 (8.7)	2.00 (1.6)	8.169	0.004273	6.818 (1.518–30.619)

*IL10* loci for haplotype analysis: rs3024498, rs1554286, rs1518111, rs1518110, rs3021094, rs1800872, rs1800896, rs1800893, rs6693899, and rs6703630.

**Table 4:** Asso + ciations between SNPs and clinical responses.

Gene	rs ID	Geno-Type	Outcome Measure	Frequency (%)		χ^2^ Value	Corrected *p*-Value	OR (95% CI)
			≤Median vs. >Median	Cases	Controls			
*IL1B*	rs16944	AA	Severity	65.4	88.4			Reference
		**GG**		**34.6**	**11.6**	**3.968**	**0.046**	**4.02** **(1.17, 13.82)**
*IL12B*	rs2569254	CC	Mode of wear	23.1	58.7			Reference
		**CT**		**76.9**	**41.3**	**4.158**	**0.041**	**4.74** **(1.19, 18.94)**
		CC	Hospital admission	35.7	74.6			Reference
		**CT**		**64.3**	**25.4**	**6.359**	**0.012**	**5.29** **(1.56, 18.0)**
		CC	Photophobia	21.4	61.2			Reference
		**CT**		**78.6**	**38.8**	**5.864**	**0.015**	**5.78** **(1.47, 22.71)**
	rs730691	TT	Mode of wear	66.7	92.7			Reference
		**TC**		**33.3**	**7.3**	**6.902**	**0.009**	**6.37** **(1.69, 23.99)**
		TT	Blurred vision	33.3	60.3			Reference
		**TC**		**66.7**	**39.7**	**3.942**	**0.047**	**3.04** **(1.12, 8.26)**
		TT	Discharge	90.9	59.6			Reference
		**TC**		**9.1**	**40.4**	**5.798**	**0.016**	**0.19** **(0.04, 0.87)**
		TT	Anterior chamber reaction	70.0	93.0			Reference
		**TC**		**30.0**	**7.0**	**5.036**	**0.025**	**5.68** **(1.41, 22.93)**

## Data Availability

The data presented in this study are available on request from the corresponding author. The data are not publicly available due to ethical issues.

## Supplementary Materials

The following supporting information can be downloaded at: https://www.mdpi.com/article/10.3390/pathogens11111387/s1, Figure S1: Location of SNPs within the interleukin genes; Supplementary Table S1: Candidate SNPs that were screened with their reliability values; Supplementary Table S2: Linkage disequilibrium between adjacent SNPs in each of the candidate genes; Supplementary Table S3: Differences in the frequency of interleukin SNPs for all cases versus controls. Supplementary Table S4: Differences in the frequency of interleukin SNPs for MK only versus controls. Supplementary Tabel S5: Differences in the frequency of interleukin SNPs for SK only versus controls. Supplementary Table S6: Differences in the frequency of interleukin SNPs for MK only versus SK only.
