# Supplementary material for: Association of Single-Nucleotide Polymorphisms in Interleukin Genes with Microbial Keratitis in a South Indian Population
Source: Pathogens. 2022 Nov 20;11(11):1387. doi: 10.3390/pathogens11111387 (PMC9692714; doi:10.3390/pathogens11111387)
Supplement: Supplementary file 1 [file pathogens-11-01387-s001.zip › pathogens-2020443-supplementary.pdf]

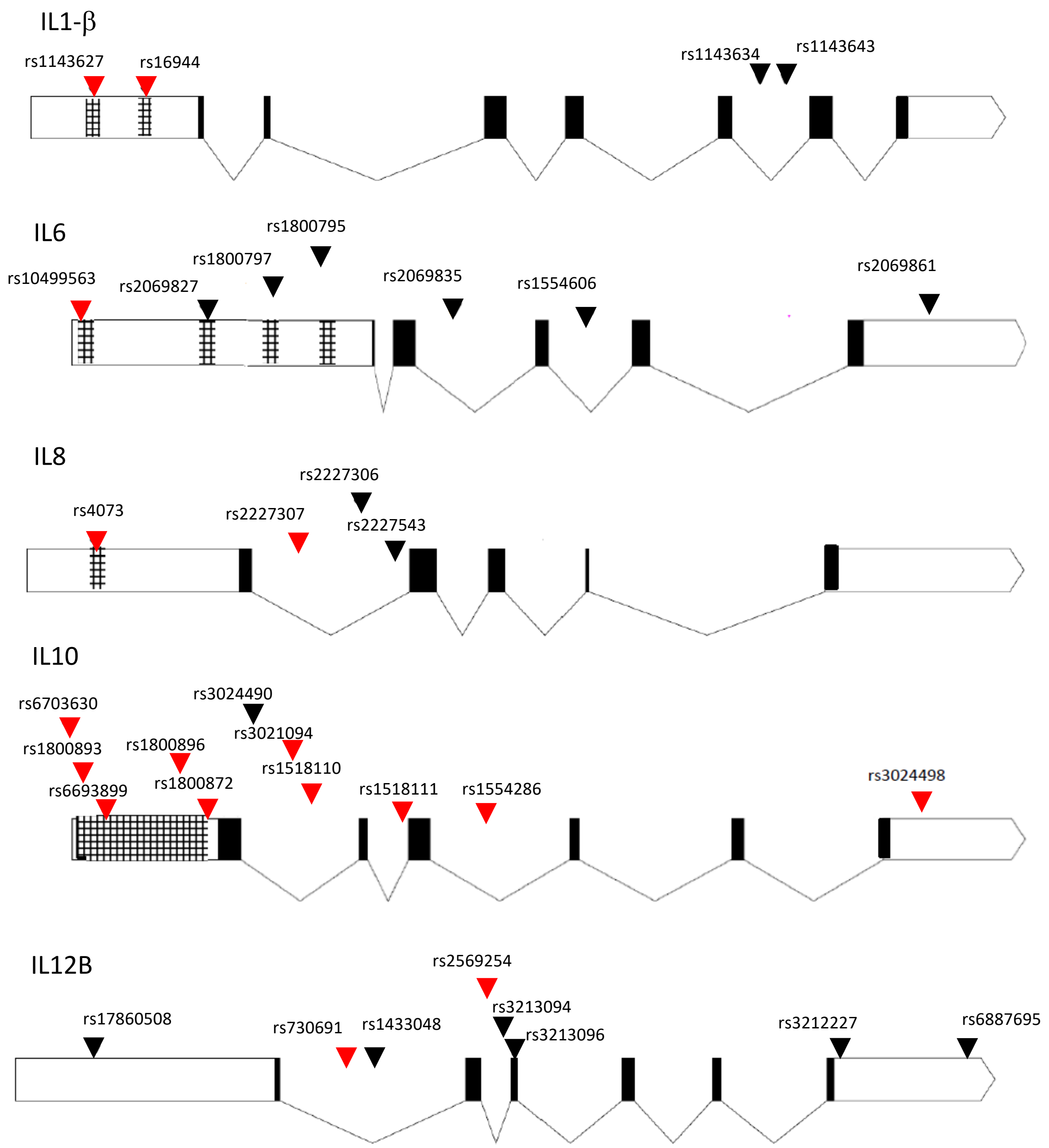

= Promoter region; = 5”-Untranslated region (UTR); = 3”-UTR;

= Exon; ______ = Intron; = Location of the SNP; = location of SNP associated with disease

Figure S1. Location of SNPs within the interleukin genes.

Supplementary Table S1. Candidate SNPs that were screened with their reliability values.

Gene name	rs id	Genomic change	Average GenTrain score	Average call rate score
IL1B				
	rs1143643	C>T	0.77	0.99
	rs78226748	C>A	0.85	0.99
	rs1143634	G>A	0.76	0.99
	rs1143627	G>A	0.82	0.99
	rs16944	A>G	0.85	0.99
IL6				
	rs10499563	T>C	0.77	0.99
	rs2069827	G>T	0.74	0.99
	rs1800797	A>G	0.70	0.99
	rs1800795	C>G	0.89	0.99
	rs2069835	T>C	0.8	1.00
	rs1554606	T>G	0.80	0.99
	rs11544633	T>C	0.81	1.00
	rs34012176	dupC	0.82	0.99
	rs2069861	C>T	0.74	1.00
IL8				
	rs2227532	T>C	0.85	0.99
	rs4073	A>T	0.88	0.99
	rs2227307	G>T	0.72	0.99
	rs2227306	C>T	0.69	0.98
	rs2227543	C>T	0.84	0.98
IL10				
	rs3024498	T>C	0.76	0.99
	rs1554286	A>G	0.81	0.99
	rs1518111	T>C	0.74	0.99
	rs1518110	A>C	0.72	0.99
	rs3021094	T>G	0.87	0.99
	rs1800872	T>G	0.83	0.99
	rs1800896	T>C	0.85	0.98
	rs1800893	C>T	0.68	0.74
	*rs6693899	G>T	NA	NA
	*rs6703630	C>T	NA	NA
IL12B				
	rs3212227	T>G	0.75	0.99
	rs3213119	C>A	0.76	0.98
	rs74644143	C>T	0.85	0.78
	rs79446920	C>T	0.75	0.99
	rs10045130	C>T	0.80	0.99
	rs3213096	C>T	0.81	0.99
	rs3213094	C>T	0.80	1.00
	rs2569254	C>T	0.84	0.99
	rs1433048	G>A	0.74	0.99
	rs730691	T>C	0.73	0.99
	rs17860508	TTAGAG>GC	0.64	0.99
	rs6887695	G>C	0.85	1.00

*SNPs rs6693899 and rs6703630 were screened using sequencing on an automated DNA sequencer rather than the GoldenGate system and so lack GenTrain and Call rates. The SNPs highlighted in grey cells were either monomorphic (i.e. no-one had variations in the screened population) or had improper data clustering in the GoldenGate® assay (IL12B rs3213096 only) and so were excluded from further analysis. IL - interleukin; rs id - Reference SNP cluster identification; GenTrain score was computed from the GenTrain 2.0 clustering algorithm; Call rate score = the ratio of a number of genotypes exceeding the threshold value to the total number of genotypes. Both GenTrain and Call rate scores were provided by the manufacturer Illumina. A GenTrain score or Call Rate of above 0.7 usually indicates well-behaving genotypes.

Supplementary Table S2. Linkage disequilibrium between adjacent SNPs in each of the candidate genes.

SNP1	SNP2	Linkage disequilibrium (D’)
**IL-1β**		
rs1143643	rs1143634	1
	rs1143627	0.96
	rs16944	0.96
rs1143634	rs1143627	0.71
	rs16944	0.7
rs1143627	rs16944	1
IL-6		
rs10499563	rs2069827	1
	rs1800797	1
	rs1800795	1
	rs2069835	1
	rs1554606	0.77
	rs2069861	1
rs2069827	rs1800797	1
	rs1800795	1
	rs2069835	1
	rs1554606	1
	rs2069861	1
rs1800797	rs1800795	0.97
	rs2069835	1
	rs1554606	0.97
	rs2069861	1
rs1800795	rs2069835	1
	rs1554606	1
	rs2069861	1
rs2069835	rs1554606	1
	rs2069861	1
rs1554606	rs2069861	1
**IL-8**		
rs4073	rs2227307	1
	rs2227306	1
	rs2227543	1
rs2227307	rs2227306	1
	rs2227543	1
rs2227306	rs2227543	1
**IL-10**		
rs3024498	rs1554286	0.96
	rs1518111	1
	rs1518110	1
	rs3021094	1
	rs1800872	1
	rs1800896	1
	rs1800893	1
	rs6693899	0.87
	rs6703630	0.96

rs1554286	rs1518111	1
	rs1518110	1
	rs3021094	0.65
	rs1800872	1
	rs1800896	1
	rs1800893	1
	rs6693899	1
	rs6703630	1
rs1518111	rs1518110	0.97
	rs3021094	0.61
	rs1800872	1
	rs1800896	1
	rs1800893	1
	rs6693899	1
	rs6703630	1
rs1518110	rs3021094	0.63
	rs1800872	0.98
	rs1800896	1
	rs1800893	1
	rs6693899	0.93
	rs6703630	1
rs3021094	rs1800872	1
	rs1800896	1
	rs1800893	1
	rs6693899	1
	rs6703630	1
	rs1800872	1
rs1800872	rs1800893	1
	rs6693899	1
	rs6703630	1
	rs1800893	1
rs1800896	rs6693899	1
	rs6703630	1
	rs6693899	1
rs1800893	rs6703630	1
	rs6703630	1
rs6693899		
**IL-12B**	rs3213094	1
rs3212227	rs2569254	1
	rs1433048	1
	rs730691	0.85
	rs17860508	0.92
	rs6887695	0.82
	rs2569254	1
rs3213094	rs1433048	1
	rs730691	0.86
	rs17860508	0.92
	rs6887695	0.63
	rs1433048	0.79

rs2569254	rs730691	1
	rs17860508	0.07
	rs6887695	0.51
	rs730691	0.79
rs1433048	rs17860508	0.15
	rs6887695	0.51
	rs17860508	1
rs730691	rs6887695	0.61
	rs6887695	1

Supplementary Table S3. Differences in the frequency of interleukin SNPs for all cases versus controls.

Gene	rs ID	Allele / Genotype	Frequency (%)		χ^2 value	Corrected P value	OR (95% CI)
			Cases (N=145)	Controls (N=189)			
IL1β	rs1143643	C	71.7	77.2			Reference
		T	28.3	22.8	0.1998	0.2	0.75 (0.45, 1.23)
		CC	51.7	58.7			Reference
		CT	39.3	36.5	0.743	0.3796	1.223 (0.774, 1.931)
		TT	9	4.8	2.744	0.1079	2.138 (0.870, 5.253)
	rs1143634	G	86.9	84.1			Reference
		A	13.1	15.9	0.317	0.4236	1.25 (0.67, 2.33)
		GG	75.2	71.4			Reference
		GA	22.8	24.9	0.286	0.5964	0.870 (0.521, 1.451)
		AA	2.1	3.7	0.814	0.3686	0.531 (0.134, 2.101)
	rs1143627	G	59.3	64			Reference
		A	40.7	36	1.392	0.4565	0.82 (0.52, 1.28)
		GG	37.9	38.6			Reference
		GA	42.1	50.8	0.494	0.4835	0.843 (0.525, 1.356)
		AA	***20***	***10.6***	***3.683***	***0.0639***	***1.925 (0.986, 3.756)***
	rs16944	A	58.6	64			Reference
		G	41.4	36	1.343	0.3107	1.26 (0.81, 1.96)
		AA	37.2	38.6			Reference
		AG	42.8	51.3	0.364	0.5325	0.864 (0.538, 1.389)
		**GG**	**20**	**10.1**	**4.397**	**0.036**	**2.063 (1.048, 4.061)**
IL6	rs10499563	T	80	81.5			Reference
		C	20	18.5	0.034	0.7792	1.10 (0.64, 1.90)
		TT	65.5	64.6			Reference
		TC	29.7	33.9	0.378	0.5574	0.863 (0.539, 1.381_
		CC	4.8	1.6	2.434	0.1019	2.996 (0.755, 11.897)
	rs2069827	G	96.6	96.8			Reference
		T	3.4	3.2	0.019	0.89	1.089 (0.326, 3.642)
		GG	93.1	94.2			Reference
		GT	6.9	5.8	0.161	0.6623	1.199 (0.495, 2.905)
		TT	0	0	-	-	-
	rs1800797	G	86.9	85.7			Reference
		A	13.1	14.3	0.097	0.8921	1.11 (0.59, 2.08)
		GG	77.2	73			Reference
		AG	19.3	25.4	0.27	0.2218	0.719 (0.424, 1.219)
		AA	3.4	1.6	0.741	0.3926	2.054 (0.480, 8.790)
	rs1800795	G	88.3	85.2			Reference

		C	11.7	14.8	0.67	0.4136	0.76 (0.40, 1.46)
		GG	80	72			Reference
		**GC**	**16.6**	**26.5**	**4.257**	**0.046**	**0.563 (0.326, 0.972)**
		CC	3.4	1.6	0.817	0.4535	1.954 (0.457, 8.352)
	rs2069835	T	93.8	95.8			Reference
		C	6.2	4.2	0.654	0.6314	1.32 (0.51, 3.42)
		TT	89.7	91			Reference
		TC	9	9	0.001	0.8981	1.012 (0.474, 2.157)
		CC	1.4	0	-	-	-
	rs1554606	G	82.1	82			Reference
		T	17.9	18	0	0.8881	1.00 (0.57, 1.76)
		GG	70.3	66.7			Reference
		GT	23.4	30.7	1.618	0.1888	0.724 (0.440, 1.191)
		TT	6.2	2.6	1.942	0.1678	2.224 (0.723, 6.842)
	rs2069861	C	98.6	98.4			Reference
		T	1.4	1.6	0.024	0.877	0.867 (0.143, 5.259)
		CC	97.2	97.4			Reference
		CT	2.8	2.6	0.004	0.95	1.044 (0.275, 3.959)
		TT	0	0	-	-	-
IL8	rs4073	A	64.8	59.3			Reference
		T	35.2	40.7	0.605	0.3147	0.79 (0.50, 1.23)
		AA	39.3	37.6			Reference
		AT	51.7	43.9	0.245	0.6144	1.126 (0.705, 1.797)
		**TT**	**9**	**18.5**	**4.333**	**0.041**	**0.463 (0.224, 0.956)**
	rs2227307	T	66.9	64			Reference
		G	33.1	36	0.102	0.5794	0.88 (0.56, 1.39)
		TT	41.4	43.4			Reference
		TG	51	41.3	1.222	0.2777	1.297 (0.818, 2.055)
		GG	***7.6***	***15.3***	***2.799***	***0.0929***	***0.518 (0.240, 1.119)***
	rs2227306	C	79.3	77.8			Reference
		T	20.7	22.2	0.803	0.6893	1.10 (0.65, 1.86)
		CC	62.1	63			Reference
		CT	34.5	29.6	0.48	0.4975	1.181 (0.738, 1.888)
		TT	3.4	7.4	1.935	0.1598	0.472 (0.164, 1.359)
	rs2227543	C	70.3	66.1			Reference
		T	29.7	33.9	0.046	0.4156	1.21 (0.76, 1.94)
		CC	46.9	46			Reference
		CT	46.2	40.2	0.267	0.6134	1.128 (0.714, 1.781)
		TT	***6.9***	***13.8***	***3.054***	***0.0879***	***0.492 (0.222, 1.090)***
IL10	rs3024498	T	90.3	91			Reference
		C	9.7	9	0.042	0.6863	1.08 (0.51, 2.27)

		TT	82.8	82.5			Reference
		TC	15.2	16.9	0.138	0.7423	0.894 (0.494, 1.617)
		CC	2.1	0.5	1.374	0.1379	3.900 (0.401, 37.96)
	rs1554286	G	56.6	58.2			Reference
		A	43.2	41.8	0.006	0.8262	0.93 (0.60, 1.45)
		GG	34.5	35.4			Reference
		GA	43.5	45	0.001	0.977	0.993 (0.608, 1.622)
		AA	22.1	19.6	0.233	0.6503	1.159 (0.637, 2.108)
	rs1518111	C	52.4	56.1			Reference
		T	47.6	43.9	0.326	0.5335	0.86 (0.56, 1.33)
		CC	31.7	31.7			Reference
		CT	41.4	48.1	0.344	0.5644	0.860 (0.520, 1.423)
		TT	26.9	20.1	0.942	0.3277	1.339 (0.743, 2.413)
	rs1518110	C	52.4	56.1			Reference
		A	47.6	43.9	0.446	0.5514	0.86 (0.56, 1.33)
		CC	30.3	31.2			Reference
		CA	44.1	49.2	0.098	0.1938	0.923 (0.558, 1.527)
		AA	25.5	19.6	0.918	0.3307	1.341 (0.736, 2.443)
	rs3021094	T	89	90.5			Reference
		G	11	9.5	2.878	0.7413	1.18 (0.58, 2.40)
		TT	80	82			Reference
		TG	18.6	17.5	0.096	0.7483	1.093 (0.623, 1.919)
		GG	1.4	0.5	0.638	0.1049	2.672 (0.239, 29.829)
	rs1800872	G	50.3	54.5			Reference
		T	49.7	45.5	0.567	0.3996	0.85 (0.55, 1.31)
		GG	27.6	30.2			Reference
		GT	45.5	48.1	0.016	0.3227	1.034 (0.747, 2.461)
		TT	26.9	21.7	0.999	0.2867	1.355 (0.747, 1.727)
	rs1800896	T	77.2	78.3			Reference
		C	22.8	21.7	0.054	0.9011	1.06 (0.63, 1.79)
		TT	60	60.8			Reference
		TC	34.5	34.9	0	0.982	1.001 (0.631, 1.588)
		CC	5.5	4.2	0.288	0.5854	1.322 (0.477, 3.661)
	rs1800893	C	75.9	78.3			Reference
		T	24.1	21.7	2.241	0.5055	0.87 (0.52, 1.46)
		CC	57.2	60.3			Reference
		CT	36.6	35.4	0.126	0.7502	1.085 (0.687, 1.718)
		TT	6.2	4.2	0.737	0.3477	1.545 (0.572, 4.173)
	rs6693899	G	78.6	83.6			Reference
		T	21.4	16.4	1.338	0.1978	1.39 (0.80, 2.41)
		GG	64.1	71.4			Reference

		GT	29	24.9	1.07	0.2967	1.297 (0.792, 2.124)
		TT	6.9	3.7	2.038	0.1518	2.074 (0.762, 5.645)
	rs6703630	C	91.7	92.1			Reference
		T	8.3	7.9	0.013	0.8432	1.05 (0.47, 2.31)
		CC	84.8	85.2			Reference
		CT	14.5	14.3	0.003	0.8901	1.018 (0.549, 1.886)
		TT	0.7	0.5	0.036	0.2767	1.309 (0.081, 21.135)
IL12B	rs3212227	T	59.66	59.79			Reference
		G	40.34	40.21	0.019	0.968	
		TT	36.8	36.5			Reference
		TG	45.5	46.6	0.056	0.7572	0.929 (0.575, 1.502)
		GG	17.7	16.9	0.09	0.7892	1.078 (0.578, 2.012)
	rs3213094	C	59.66	60.05			Reference
		T	40.34	39.95	0.003	0.953	1.013 (0.651, 1.576)
		CC	37.24	36.51			Reference
		CT	44.83	47.09	0.08	0.777	0.933 (0.578, 1.506)
		TT	17.93	16.4	0.046	0.83	1.072 (0.570, 2.015)
	rs2569254	C	90	85.71			Reference
		T	10	14.29	1.634	0.204	0.641 (0.323, 1.272)
		CC	82.76	74.07			Reference
		CT	**14.48**	**23.28**	**3.995**	**0.0446**	**0.557 (0.314, 0.989)**
		TT	2.76	2.65	0.01	0.8092	0.933 (0.245, 3.554)
	rs1433048	A	88.3	91			Reference
		G	11.7	9	0.669	0.415	0.744 (0.366, 1.514)
		AA	77.2	82.5			Reference
		GA	21.4	16.9	1.136	0.311	1.349 (0.778, 2.340)
		GG	1.4	0.5	0.693	0.27	2.786 (0.250, 31.10)
	rs730691	T	49.7	53.4			Reference
		C	50.3	46.6	0.471	0.493	1.164 (0.755, 1.795)
		TT	28.3	25.4			Reference
		**TC**	**42.8**	**57.1**	**7.973**	**0.005**	**0.451 (0.260, 0.784)**
		CC	29	17.5	1.601	0.2128	0.671 (0.362, 1.245)
	rs17860508	C	90.3	89.9			Reference
		T	9.7	10.1	0	0.999	0.95 (0.46, 1.98)
		CC	80.0	79.4			Reference
		CT	20.0	20.6	0	0.892	0.96 (0.56, 1.63)
		TT	0.0	0.0	-	-	-
	rs6887695	G	59.3	58.2			Reference
		C	40.7	41.8	0.042	0.838	0.955 (0.615, 1.483)
		GG	34.5	31.2			Reference
		GC	49.7	53.4	0.492	0.5005	0.841 (0.519, 1.364)

		CC	15.9	15.3	0.038	0.8611	0.936 (0.482, 1.819)

Data in bold type indicates significant differences (p<0.05) between cases and controls. The data in bold and italics indicates trends (P<0.1) between cases and controls. rs id - Reference SNP cluster identification; OR - Odds ratio; CI = confidence interval.

Supplementary Table S4. Differences in the frequency of interleukin SNPs for MK only versus controls.

Gene	rs ID	Allele / Genotype	Frequency (%)		X^2 value	Corrected p value	OR (95% CI)
			Cases (N=81)	Controls (N=189)			
IL1β	rs1143643	C	70.4	77.2			Reference
		T	29.6	22.8	1.44	0.297	1.43 (0.80, 2.57)
		CC	49.4	58.7			Reference
		CT	42.0	36.5	1.26	0.276	1.37 (0.79, 2.36)
		TT	8.6	4.8	1.36	0.139	2.16 (0.75, 6.18)
	rs1143634	G	88.3	84.1			Reference
		A	11.7	15.9	0.69	0.329	0.66 (0.3, 1.47)
		GG	77.8	71.4			Reference
		GA	21	24.9	0.41	0.431	0.78 (0.41, 1.46)
		AA	1.2	3.7	0.59	0.205	0.31 (0.04, 2.54)
	rs1143627	G	58.6	64			Reference
		A	41.4	36	0.36	0.486	1.22 (0.72, 2,09)
		GG	39.5	38.6			Reference
		GA	38.3	50.8	0.78	0.318	0.74 (0.41, 1.32)
		AA	***22.2***	***10.6***	***2.8***	***0.069***	***2.05 (0.96, 4.39)***
	rs16944	A	58	64			Reference
		G	42	36	0.77	0.408	1.32 (0.77, 2.24)
		AA	38.3	38.6			Reference
		AG	39.5	51.3	0.62	0.380	0.76 (0.43, 1.36)
		**GG**	**22.2**	**10.1**	**3.34**	**0.036**	**2.20 (1.02, 4.75)**
IL6	rs10499563	T	81.5	81.5			Reference
		C	18.5	18.5	0.03	1.000	1 (0.51, 1.95)
		TT	67.9	64.6			Reference
		TC	27.2	33.9	0.6	0.350	0.76 (0.43, 1.36)
		CC	***4.9***	***1.6***	***2.1***	***0.093***	***2.95 (0.64, 13.66)***
	rs2069827	G	95.1	96.8			Reference
		T	4.9	3.2	0.12	0.728	1.58 (0.43, 5.77)
		GG	90.1	94.2			Reference
		GT	9.9	5.8	0.87	0.200	1.77 (0.69, 4.59)
		TT	0.0	0	-	-	-
	rs1800797	G	86.4	85.7			Reference
		A	13.6	14.3	0.001	0.872	0.94 (0.44, 2.01)
		GG	75.3	73			Reference
		AG	21.0	25.4	0.28	0.521	0.8 (0.43, 1.50)
		AA	3.7	1.6	0.31	0.213	2.88 (0.56, 14.73)
	rs1800795	G	87.7	85.2			Reference
		C	12.3	14.8	0.12	0.578	0.81 (0.37, 1.76)

		GG	79.0	72			Reference
		GC	17.3	26.5	1.93	0.102	0.59 (0.31, 1.16)
		CC	3.7	1.6	0.24	0.265	2.13 (0.42, 10.82)
	rs2069835	T	92.6	95.8			Reference
		C	7.4	4.2	0.6	0.244	1.81 (0.61, 5.39)
		TT	86.4	91			Reference
		TC	12.3	9	0.43	0.402	1.45 (0.63, 3.31)
		CC	1.2	0	-	-	-
	rs1554606	G	82.7	82			Reference
		T	17.3	18	0.001	1.000	0.95 (0.48, 1.89)
		GG	69.1	66.7			Reference
		GT	25.9	30.7	0.28	0.481	0.82 (0.45, 1.47)
		TT	4.9	2.6	0.24	0.398	1.8 (0.47, 6.96)
	rs2069861	C	100.0	98.4			Reference
		T	0.0	1.6	-	-	-
		CC	100.0	97.4			Reference
		CT	0.0	2.6	-	-	-
		TT	0	0	-	-	-
IL8	rs4073	A	66.7	59.3			Reference
		T	33.3	40.7	1.02	0.397	0.73 (0.42, 1.26)
		AA	40.7	37.6			Reference
		AT	50.6	43.9	0.02	0.811	1.00 (0.56, 1.75)
		TT	***8.6***	***18.5***	***2.7***	***0.062***	***0.43 (0.17, 1.07)***
	rs2227307	T	67.9	64			Reference
		G	32.1	36	0.22	0.584	0.84 (0.48, 1.46)
		TT	42.0	43.4			Reference
		TG	51.9	41.3	0.63	0.346	1.3 (0.75, 2.25)
		GG	***6.2***	***15.3***	***2.21***	***0.086***	***0.42 (0.15, 1.17)***
	rs2227306	C	79.0	77.8			Reference
		T	21.0	22.2	0.004	0.862	0.93 (0.49, 1.76)
		CC	60.5	63			Reference
		CT	35.8	29.6	0.44	0.429	1.26 (0.72, 2.2)
		TT	3.7	7.4	0.523	0.339	0.52 (0.14, 1.89)
	rs2227543	C	70.4	66.1			Reference
		T	29.6	33.9	0.02	0.541	0.92 (0.52, 1.63)
		CC	46.9	46			Reference
		CT	46.9	40.2	3.3	0.117	1.81 (1, 3.29)
		TT	6.2	13.8	0.18	0.6214	0.7 (0.24, 2.01)
IL10	rs3024498	T	90.1	91			Reference
		C	9.9	9	0	0.831	1.11 (0.46, 2.68)
		TT	82.7	82.5			Reference
		TC	14.8	16.9	0.04	0.697	0.87 (0.42, 1.8)
		CC	2.5	0.5	0.543	0.102	1.16 (0.10, 13.06)

	rs1554286	G	54.3	58.2			Reference
		A	45.7	41.8	0.21	0.589	1.17 (0.69, 1.98)
		GG	32.1	35.4			Reference
		GA	43.2	45	0	0.832	1.06 (0.58, 1.93)
		AA	24.7	19.6	0.54	0.357	1.39 (0.69, 2.83)
	rs1518111	C	49.4	56.1			Reference
		T	50.6	43.9	0.77	0.270	1.31 (0.78, 2.21)
		CC	29.6	31.7			Reference
		CT	38.3	48.1	0.12	0.606	0.85 (0.46, 1.59)
		TT	32.1	20.1	1.85	0.115	1.71 (0.86, 3.4)
	rs1518110	C	49.4	56.1			Reference
		A	50.6	43.9	0.77	0.284	1.31 (0.78, 2.21)
		CC	27.2	31.2			Reference
		CA	43.2	49.2	0.02	0.999	1.01 (0.54, 1.89)
		AA	29.6	19.6	1.83	0.120	1.74 (0.86, 3.54)
	rs3021094	T	87.7	90.5			Reference
		G	12.3	9.5	0.23	0.419	1.34 (0.59, 3.04)
		TT	75.3	82			Reference
		TG	23.5	17.5	1.01	0.231	1.46 (0.77, 2.77)
		GG	1.2	0.5	0.012	0.360	2.54 (0.16, 41.27)
	rs1800872	G	46.9	54.5			Reference
		T	53.1	45.5	1.02	0.294	1.36 (0.8, 2.28)
		GG	25.9	30.2			Reference
		GT	42.0	48.1	0.01	0.999	1.01 (0.54, 1.92)
		TT	32.1	21.7	1.81	0.116	1.72 (0.85, 3.47)
	rs1800896	T	76.5	78.3			Reference
		C	23.5	21.7	0.03	0.761	1.11 (0.6, 2.06)
		TT	59.3	60.8			Reference
		TC	34.6	34.9	0.01	0.940	1.02 (0.58, 1.77)
		CC	6.2	4.2	0.135	0.467	1.5 (0.47, 4.81)
	rs1800893	C	75.3	78.3			Reference
		T	24.7	21.7	0.15	0.640	1.18 (0.64, 2.18)
		CC	56.8	60.3			Reference
		CT	37.0	35.4	0.05	0.736	1.11 (0.64, 1.92)
		TT	6.2	4.2	0.178	0.446	1.55 (0.48, 4.98)
	rs6693899	G	80.2	83.6			Reference
		T	19.8	16.4	0.24	0.620	1.26 (0.64, 2.45)
		GG	67.9	71.4			Reference
		GT	24.7	24.9	0	0.867	1.05 (0.57, 1.92)
		TT	7.4	3.7	0.993	0.177	2.10 (0.68, 6.54)
	rs6703630	C	92.6	92.1			Reference
		T	7.4	7.9	0.01	1.000	0.93 (0.35, 2.48)
		CC	86.4	85.2			Reference

		CT	12.3	14.3	0.04	0.678	0.85 (0.39, 1.86)
		TT	1.2	0.5	0.029	0.866	2.3 (0.14, 37.3)
IL12B	rs3212227	T	56.8	59.8			Reference
		G	43.2	40.2	0.1	0.486	1.13 (0.67, 1.92)
		TT	35.8	36.5			Reference
		TG	40.7	46.6	0.05	0.731	0.89 (0.49, 1.61)
		GG	23.5	16.9	0.59	0.352	1.41 (0.69, 2.89)
	rs3213094	C	56.8	60.1			Reference
		T	43.2	40.0	0.1	0.686	1.13 (0.67, 1.92)
		CC	35.8	36.5			Reference
		CT	42.0	47.1	0.03	0.768	0.91 (0.51, 1.63)
		TT	22.2	16.4	0.47	0.387	1.38 (0.67, 2.85)
	rs2569254	C	91.4	85.7			Reference
		T	8.6	14.3	1.17	0.189	0.57 (0.24, 1.36)
		CC	85.2	74.1			Reference
		CT	**12.3**	**23.3**	**3.63**	**0.045**	**0.46 (0.22, 0.97)**
		TT	2.5	2.7	0.027	0.600	0.81 (0.15, 4.29)
	rs1433048	A	87.7	91.0			Reference
		G	12.3	9.0	0.38	0.513	1.43 (0.62, 3.26)
		AA	77.8	82.5			Reference
		GA	19.8	16.9	0.21	0.523	1.24 (0.64, 2.41)
		GG	2.5	0.5	0.631	0.205	4.95 (0.44, 55.6)
	rs730691	T	53.1	53.4			Reference
		C	46.9	46.6	0.01	0.999	1.01 (0.6, 1.71)
		TT	32.1	25.4			Reference
		**TC**	**40.7**	**57.1**	**2.79**	**0.007**	**0.56 (0.31, 1.05)**
		CC	27.2	17.5	0.15	0.137	1.23 (0.6, 2.53)
	rs17860508	C	90.1	89.9			Reference
		T	9.9	10.1	0.02	0.999	1.03 (0.43, 2.46)
		CC	80.2	79.4			Reference
		CT	19.8	20.6	0	1.000	0.95 (0.49, 1.82)
		TT	0.0	0.0	-	-	-
	rs6887695	G	60.5	58.2			Reference
		C	39.5	41.8	0.05	0.892	0.91 (0.53, 1.55)
		GG	35.8	31.2			Reference
		GC	48.1	53.4	0.45	0.406	0.79 (0.44, 1.4)
		CC	16.0	15.3	0	0.8172	0.91 (0.41, 2.01)

Data in bold type indicates significant differences ($p<0.05$) between cases and controls. Data in bold and italics indicates a trend ($p<0.1$) for a difference between cases and controls. rs id - Reference SNP cluster identification; OR - Odds ratio; CI = confidence interval

Supplementary Table S5. Differences in the frequency of interleukin SNPs for SK only versus controls.

Gene	rs ID	Allele / Genotype	Frequency (%)		X^2 value	Corrected p value	OR (95% CI)
			Cases (N=64)	Controls (N=189)			
IL1β	rs1143643	C	73.4	77.2			Reference
		T	26.6	22.8	0.04	0.583	1.12 (0.59, 2.14)
		CC	54.7	58.7			Reference
		CT	35.9	36.5	0	0.827	1.06 (0.58, 1.94)
		TT	9.4	4.8	1.09	0.126	2.11 (0.70, 6.36)
	rs1143634	G	84.4	84.1			Reference
		A	15.6	15.9	0.02	0.829	0.98 (0.45, 2.14)
		GG	71.9	71.4			Reference
		GA	25.0	24.9	0.03	0.960	1 (0.52, 1.93)
		AA	3.1	3.7	0.016	0.749	0.84 (0.17, 4.18)
	rs1143627	G	59.4	64			Reference
		A	40.6	36	0.27	0.540	1.22 (0.68, 2.18)
		GG	35.9	38.6			Reference
		GA	46.9	50.8	0.02	0.975	0.99 (0.53, 1.85)
		AA	17.2	10.6	1.05	0.216	1.75 (0.73, 4.18)
	rs16944	A	59.4	64			Reference
		G	40.6	36	0.27	0.518	1.22 (0.68, 2.18)
		AA	35.9	38.6			Reference
		AG	46.9	51.3	0.01	0.962	0.98 (0.53, 1.83)
		GG	17.2	10.1	1.28	0.1628	1.84 (0.76, 4.42)
IL6	rs10499563	T	79.7	81.5			Reference
		C	20.3	18.5	0.02	0.569	1.12 (0.55, 2.28)
		TT	62.5	64.6			Reference
		TC	32.8	33.9	0.02	0.983	1 (0.55, 1.84)
		CC	4.7	1.6	0.84	0.112	3.05 (0.59, 15.72)
	rs2069827	G	98.4	96.8			Reference
		T	1.6	3.2	0.05	0.682	0.48 (0.06, 4.1)
		GG	96.9	94.2			Reference
		GT	3.1	5.8	0.27	0.523	0.52 (0.11, 2.42)
		TT	0.0	0	-	-	-
	rs1800797	G	89.1	85.7			Reference
		A	10.9	14.3	0.22	0.538	0.74 (0.30, 1.78)
		GG	79.7	73			Reference
		AG	17.2	25.4	1.25	0.213	0.62 (0.3, 1.29)
		AA	3.1	1.6	0.019	0.324	1.80 (0.29, 11.11)
	rs1800795	G	89.1	85.2			Reference
		C	10.9	14.8	0.32	0.419	0.71 (0.29, 1.71)

		GG	81.3	72			Reference
		GC	15.6	26.5	2.37	0.113	0.52 (0.25, 1.11)
		CC	3.1	1.6	0.01	0.340	1.74 (0.28, 10.74)
	rs2069835	T	96.9	95.8			Reference
		C	3.1	4.2	0	1.000	0.73 (0.15, 3.53)
		TT	93.8	91			Reference
		TC	4.7	9	0.65	0.300	0.51 (0.14, 1.79)
		CC	1.6	0	-	-	-
	rs1554606	G	82.8	82			Reference
		T	17.2	18	0	1.000	0.95 (0.45, 2.0)
		GG	71.9	66.7			Reference
		GT	20.3	30.7	1.51	0.164	0.61 (0.31, 1.22)
		TT	***7.8***	***2.6***	***1.51***	***0.090***	***2.74 (0.76, 9.9)***
	rs2069861	C	96.9	98.4			Reference
		T	3.1	1.6	0.06	0.603	2.00 (0.33, 12.25)
		CC	93.8	97.4			Reference
		CT	6.3	2.6	0.912	0.236	2.45 (0.64, 9.43)
		TT	0	0	-	-	-
IL8	rs4073	A	64.1	59.3			Reference
		T	35.9	40.7	0.28	0.565	0.82 (0.45, 1.47)
		AA	37.5	37.6			Reference
		AT	53.1	43.9	0.21	0.532	1.21 (0.66, 2.23)
		TT	9.4	18.5	1.31	0.164	0.51 (0.19, 1.35)
	rs2227307	T	65.6	64			Reference
		G	34.4	36	0.01	0.767	0.93 (0.51, 1.69)
		TT	40.6	43.4			Reference
		TG	50.0	41.3	0.47	0.448	1.29 (0.71, 2.37)
		GG	9.4	15.3	0.39	0.421	0.65 (0.24, 1.75)
	rs2227306	C	81.3	77.8			Reference
		T	18.8	22.2	0.17	0.621	0.81 (0.4, 1.65)
		CC	64.1	63			Reference
		CT	32.8	29.6	0.01	0.764	1.09 (0.59, 2.01)
		TT	3.1	7.4	0.739	0.257	0.42 (0.09, 1.90)
	rs2227543	C	70.3	66.1			Reference
		T	29.7	33.9	0.21	0.639	0.83 (0.45, 1.53)
		CC	46.9	46			Reference
		CT	45.3	40.2	0.03	0.712	1.11 (0.61, 2.01)
		TT	7.81	13.8	0.76	0.252	0.56 (0.2, 1.58)
IL10	rs3024498	T	90.6	91			Reference
		C	9.4	9	0.03	0.999	1.05 (0.39, 2.78)
		TT	82.8	82.5			Reference
		TC	15.6	16.9	0.06	0.794	0.84 (0.39, 1.82)
		CC	1.6	0.5	0	0.192	2.69 (0.17, 43.71)

	rs1554286	G	59.4	58.2			Reference
		A	40.6	41.8	0	0.867	0.95 (0.53, 1.7)
		GG	37.5	35.4			Reference
		GA	43.8	45	0.01	0.790	0.92 (0.49, 1.73)
		AA	18.8	19.6	0	0.804	0.91 (0.41, 2.02)
	rs1518111	C	57.8	56.1			Reference
		T	42.2	43.9	0.01	0.871	0.93 (0.53, 1.65)
		CC	34.4	31.7			Reference
		CT	45.3	48.1	0.07	0.668	0.87 (0.46, 1.65)
		TT	20.3	20.1	0	0.867	0.93 (0.42, 2.07)
	rs1518110	C	57.8	56.1			Reference
		A	42.2	43.9	0.01	0.883	0.93 (0.53, 1.65)
		CC	34.4	31.2			Reference
		CA	45.3	49.2	0.14	0.746	0.84 (0.44, 1.59)
		AA	20.3	19.6	0	0.897	0.94 (0.42, 2.1)
	rs3021094	T	92.2	90.5			Reference
		G	7.8	9.5	0.03	0.786	0.81 (0.29, 2.26)
		TT	85.9	82			Reference
		TG	12.5	17.5	0.5	0.426	0.68 (0.3, 1.57)
		GG	1.6	0.5	0.002	0.340	2.82 (0.17, 45.83)
	rs1800872	G	54.7	54.5			Reference
		T	45.3	45.5	0.01	0.896	0.99 (0.56, 1.75)
		GG	29.7	30.2			Reference
		GT	50.0	48.1	0	0.783	1.06 (0.55, 2.04)
		TT	20.3	21.7	0.01	0.909	0.95 (0.42, 2.14)
	rs1800896	T	78.1	78.3			Reference
		C	21.9	21.7	0.02	1.000	1.01 (0.51, 2.01)
		TT	60.9	60.8			Reference
		TC	34.4	34.9	0.01	0.977	0.98 (0.54, 1.8)
		CC	4.7	4.2	0.046	0.838	1.11 (0.28, 4.38)
	rs1800893	C	76.6	78.3			Reference
		T	23.4	21.7	0.01	0.760	1.11 (0.56, 2.17)
		CC	57.8	60.3			Reference
		CT	35.9	35.4	0	0.834	1.06 (0.58, 1.93)
		TT	6.3	4.2	0.111	0.523	1.54 (0.44, 5.41)
	rs6693899	G	76.6	83.6			Reference
		T	23.4	16.4	1.15	0.261	1.56 (0.78, 3.13)
		GG	59.4	71.4			Reference
		GT	34.4	24.9	2.1	0.101	1.66 (0.89, 3.1)
		TT	6.3	3.7	0.537	0.272	2.03 (0.56, 7.30)
	rs6703630	C	92.2	92.1			Reference
		T	7.8	7.9	0.06	0.616	0.98 (0.34, 2.82)
		CC	82.8	85.2			Reference

		CT	17.2	14.3	0.12	0.610	1.24 (0.58, 2.66)
		TT	0	0.5	-	-	-
IL12B	rs3212227	T	64.1	59.8			Reference
		G	35.9	40.2	0.21	0.557	0.83 (0.46, 1.50)
		TT	39.1	36.5			Reference
		TG	48.4	46.6	0	1.000	0.97 (0.53, 1.8)
		GG	12.5	16.9	0.35	0.513	0.69 (0.28, 1.7)
	rs3213094	C	64.1	60.1			Reference
		T	35.9	40.0	0.21	0.557	0.83 (0.46, 1.50)
		CC	39.1	36.5			Reference
		CT	48.4	47.1	0	1.000	0.96 (0.52, 1.78)
		TT	12.5	16.4	0.27	0.515	0.71 (0.29, 1.76)
	rs2569254	C	89.1	85.7			Reference
		T	10.9	14.3	0.22	0.535	0.74 (0.3, 1.78)
		CC	79.7	74.1			Reference
		CT	17.2	23.3	0.69	0.379	0.69 (0.33, 1.43)
		TT	3.1	2.7	0.105	1.000	1.1 (0.21, 5.84)
	rs1433048	A	87.5	91.0			Reference
		G	12.5	9.0	0.32	0.467	1.45 (0.59, 3.53)
		AA	76.6	82.5			Reference
		GA	21.9	16.9	0.54	0.451	1.39 (0.69, 2.82)
		GG	1.6	0.5	0	0.425	3.18 (0.2, 51.85)
	rs730691	T	48.4	53.4			Reference
		C	51.6	46.6	0.3	0.562	1.22 (0.69, 2.16)
		TT	25.0	25.4			Reference
		TC	45.3	57.1	0.18	0.587	0.81 (0.40, 1.62)
		CC	29.7	17.5	1.31	0.223	1.73 (0.78, 3.84)
	rs17860508	C	90.6	89.9			Reference
		T	9.4	10.1	0.01	1.000	0.93 (0.35, 2.43)
		CC	79.7	79.4			Reference
		CT	20.3	20.6	0.02	1.000	0.98 (0.49, 1.98)
		TT	0.0	0.0	-	-	-
	rs6887695	G	59.4	58.2			Reference
		C	40.6	41.8	0	0.884	0.95 (0.54, 1.7)
		GG	32.8	31.2			Reference
		GC	51.6	53.4	0.01	0.871	0.92 (0.49, 1.73)
		CC	15.63	15.3	0.02	1	0.97 (0.40, 2.32)

Data in bold type indicates significant differences (p<0.05) between cases and controls. Data in bold and italics indicates a trend (p<0.1) for a difference between cases and controls. rs id - Reference SNP cluster identification; OR - Odds ratio; CI = confidence interval

Supplementary Table S6. Differences in the frequency of interleukin SNPs for MK only versus SK only.

Gene	rs ID	Allele / Genotype	Frequency (%)		X^2 value	Corrected p value	OR (95% CI)
			MK Cases (N=81)	SK Cases (N=64)			
IL1b	rs1143643	C	70.4	73.4			Reference
		T	29.6	26.6	0.05	0.714	1.16 (0.56, 2.42)
		CC	49.4	54.7			Reference
		CT	42.0	35.9	0.3	0.484	1.29 (0.64, 2.6)
		TT	8.6	9.4	0.07	1.000	1.02 (0.31, 3.33)
	rs1143634	G	88.3	84.4			Reference
		A	11.7	15.6	0.3	0.464	0.68 (0.26, 1.78)
		GG	77.8	71.9			Reference
		GA	21.0	25.0	0.19	0.552	0.78 (0.36, 1.7)
		AA	1.2	3.1	0.064	0.799	0.37 (0.03, 4.15)
	rs1143627	G	58.6	59.4			Reference
		A	41.4	40.6	0	1.000	1.06 (0.54, 2.06)
		GG	39.5	35.9			Reference
		GA	38.3	46.9	0.37	0.459	0.74 (0.36, 1.55)
		AA	22.2	17.2	0.01	0.817	1.18 (0.47, 2.96)
	rs16944	A	58.0	59.4			Reference
		G	42.0	40.6	0	1.000	1.06 (0.54, 2.06)
		AA	38.3	35.9			Reference
		AG	39.5	46.9	0.19	0.578	0.79 (0.38, 1.65)
		GG	22.2	17.2	0.03	0.815	1.21 (0.48, 3.06)
IL6	rs10499563	T	81.5	79.7			Reference
		C	18.5	20.3	0	0.834	0.89 (0.39, 2.04)
		TT	67.9	62.5			Reference
		TC	27.2	32.8	0.310	0.579	0.76 (0.37, 1.57)
		CC	4.9	4.7	0.127	0.721	0.55 (0.12, 2.57)
	rs2069827	G	95.1	98.4			Reference
		T	4.9	1.6	0.419	0.517	3.27 (0.36, 30.03)
		GG	90.1	96.9			Reference
		GT	9.9	3.1	1.595	0.206	3.4 (0.7, 16.6)
		TT	0.0	0.0	-	-	-
	rs1800797	G	86.4	89.1			Reference
		A	13.6	10.9	0.05	0.800	1.28 (0.47, 3.51)
		GG	75.3	79.7			Reference
		AG	21.0	17.2	0.150	0.672	1.29 (0.56, 3.01)
		AA	3.7	3.1	0.046	0.829	1.25 (0.20, 7.8)
	rs1800795	G	87.7	89.1			Reference
		C	12.3	10.9	0	1.000	1.15 (0.41, 3.20)
		GG	79.0	81.3			Reference

		GC	17.3	15.6	0.000	0.824	1.14 (0.47, 2.77)
		CC	3.7	3.1	0.06	0.805	1.22 (0.2, 7.57)
	rs2069835	T	92.6	96.9			Reference
		C	7.4	3.1	0.57	0.450	2.48 (0.48, 12.73)
		TT	86.4	93.8			Reference
		TC	12.3	4.7	1.700	0.146	2.86 (0.75, 10.86)
		CC	1.2	1.6	0.367	0.544	0.86 (0.05, 14.00)
	rs1554606	G	82.7	82.8			Reference
		T	17.3	17.2	0.04	1.000	1.01 (0.42, 2.4)
		GG	69.1	71.9			Reference
		GT	25.9	20.3	0.25	0.551	1.33 (0.6, 2.94)
		TT	4.9	7.8	0.064	0.799	0.66 (0.17, 2.59)
	rs2069861	C	100.0	96.9			Reference
		T	0.0	3.1	-	-	-
		CC	100.0	93.8			Reference
		CT	0.0	6.3	-	-	-
		TT	0.0	0.0	-	-	-
IL8	rs4073	A	66.7	64.1			Reference
		T	33.3	35.9	0.02	0.860	0.89 (0.45, 1.78)
		AA	40.7	37.5			Reference
		AT	50.6	53.1	0.04	0.726	0.88 (0.44, 1.76)
		TT	8.6	9.4	0	1.000	0.85 (0.25, 2.85)
	rs2227307	T	67.9	65.6			Reference
		G	32.1	34.4	0.01	0.859	0.90 (0.45, 1.81)
		TT	42.0	40.6			Reference
		TG	51.9	50.0	0.03	1.000	1.00 (0.51, 2.00)
		GG	6.2	9.4	0.127	0.720	0.64 (0.18, 2.32)
	rs2227306	C	79.0	81.3			Reference
		T	21.0	18.8	0.02	0.835	1.15 (0.51, 2.63)
		CC	60.5	64.1			Reference
		CT	35.8	32.8	0.05	0.725	1.16 (0.58, 2.32)
		TT	3.7	3.1	0.047	0.826	1.26 (0.2, 7.88)
	rs2227543	C	70.4	70.3			Reference
		T	29.6	29.7	0.03	1.000	1.00 (0.49, 2.04)
		CC	46.9	46.9			Reference
		CT	46.9	45.3	0.01	1.000	1.04 (0.52, 2.04)
		TT	6.2	7.8	0	0.993	0.79 (0.21, 2.98)
IL10	rs3024498	T	90.1	90.6			Reference
		C	9.9	9.4	0.03	1.000	1.06 (0.35, 3.23)
		TT	82.7	82.8			Reference
		TC	14.8	15.6	0.01	0.976	0.95 (0.38, 2.37)
		CC	2.5	1.6	0.046	0.829	1.58 (0.14, 17.92)

	rs1554286	G	54.3	59.4			Reference
		A	45.7	40.6	0.19	0.613	1.23 (0.63, 2.39)
		GG	32.1	37.5			Reference
		GA	43.2	43.8	0.03	0.849	1.15 (0.55, 2.43)
		AA	24.7	18.8	0.5	0.372	1.54 (0.62, 3.81)
	rs1518111	C	49.4	57.8			Reference
		T	50.6	42.2	0.71	0.321	1.41 (0.73, 2.72)
		CC	29.6	34.4			Reference
		CT	38.3	45.3	0.02	1.000	0.98 (0.45, 2.11)
		TT	32.1	20.3	1.28	0.192	1.83 (0.76, 4.43)
	rs1518110	C	49.4	57.8			Reference
		A	50.6	42.2	0.71	0.321	1.41 (0.73, 2.72)
		CC	27.2	34.4			Reference
		CA	43.2	45.3	0.08	0.696	1.21 (0.56, 2.60)
		AA	29.6	20.3	1.25	0.260	1.85 (0.75, 4.53)
	rs3021094	T	87.7	92.2			Reference
		G	12.3	7.8	0.38	0.422	1.66 (0.54, 5.13)
		TT	75.3	85.9			Reference
		TG	23.5	12.5	2.14	0.131	2.14 (0.87, 5.28)
		GG	1.2	1.6	0.411	0.521	0.90 (0.06, 14.76)
	rs1800872	G	46.9	54.7			Reference
		T	53.1	45.3	2.28	0.121	1.79 (0.91, 3.54)
		GG	25.9	29.7			Reference
		GT	42.0	50.0	0.01	1.000	0.96 (0.44, 2.11)
		TT	32.1	20.3	1.11	0.253	1.81 (0.73, 4.5)
	rs1800896	T	76.5	78.1			Reference
		C	23.5	21.9	0	0.844	1.1 (0.5, 2.4)
		TT	59.3	60.9			Reference
		TC	34.6	34.4	0.01	1.000	1.03 (0.51, 2.08)
		CC	6.2	4.7	0	0.978	1.35 (0.30, 6.02)
	rs1800893	C	75.3	76.6			Reference
		T	24.7	23.4	0	1.000	1.07 (0.5, 2.31)
		CC	56.8	57.8			Reference
		CT	37.0	35.9	0	1.000	1.05 (0.52, 2.10)
		TT	6.2	6.3	0.119	0.729	1.01 (0.25, 4.01)
	rs6693899	G	80.2	76.6			Reference
		T	19.8	23.4	0.11	0.684	0.80 (0.36, 1.78)
		GG	67.9	59.4			Reference
		GT	24.7	34.4	1.12	0.262	0.63 (0.30, 1.31)
		TT	7.4	6.3	0.081	0.774	1.04 (0.27, 3.92)
	rs6703630	C	92.6	92.2			Reference
		T	7.4	7.8	0.05	0.822	0.94 (0.28, 3.25)
		CC	86.4	82.8			Reference

		CT	12.3	17.2	0.31	0.481	0.69 (0.27, 1.74)
		TT	1.2	0.0	-	-	-
IL12B	rs3212227	T	56.8	64.1			Reference
		G	43.2	35.9	0.51	0.398	1.36 (0.69, 2.66)
		TT	35.8	39.1			Reference
		TG	40.7	48.4	0	0.854	0.92 (0.44, 1.9)
		GG	23.5	12.5	1.44	0.230	2.05 (0.77, 5.48)
	rs3213094	C	56.8	64.1			Reference
		T	43.2	35.9	0.51	0.398	1.36 (0.69, 2.66)
		CC	35.8	39.1			Reference
		CT	42.0	48.4	0	1.000	0.95 (0.46, 1.95)
		TT	22.2	12.5	1.16	0.229	1.94 (0.72, 5.22)
	rs2569254	C	91.4	89.1			Reference
		T	8.6	10.9	0.03	0.778	0.77 (0.26, 2.32)
		CC	85.2	79.7			Reference
		CT	12.3	17.2	0.36	0.477	0.67 (0.27, 1.70)
		TT	2.5	3.1	0.046	0.820	0.74 (0.1, 5.42)
	rs1433048	A	87.7	87.5			Reference
		G	12.3	12.5	0.05	1.000	0.99 (0.36, 2.66)
		AA	77.8	76.6			Reference
		GA	19.8	21.9	0.01	0.837	0.89 (0.40, 2.00)
		GG	2.5	1.6	0.053	0.817	1.56 (0.14, 17.66)
	rs730691	T	53.1	48.4			Reference
		C	46.9	51.6	0.15	0.618	0.83 (0.43, 1.60)
		TT	32.1	25.0			Reference
		**TC**	40.7	45.3	0.46	0.424	0.70 (0.32, 1.56)
		CC	27.2	29.7	0.29	0.508	0.71 (0.3, 1.71)
	rs17860508	C	90.1	90.6			Reference
		T	9.9	9.4	0.03	1.000	1.06 (0.35, 3.23)
		CC	80.2	79.7			Reference
		CT	19.8	20.3	0.02	1.000	0.97 (0.43, 2.19)
		TT	0.0	0.0	-	-	-
	rs6887695	G	60.5	59.4			Reference
		C	39.5	40.6	0	1.000	0.96 (0.49, 1.86)
		GG	35.8	32.8			Reference
		GC	48.1	51.6	0.05	0.713	0.86 (0.41, 1.77)
		CC	16.0	15.6	0.02	1.000	0.94 (0.35, 2.55)

rs id - Reference SNP cluster identification; OR - Odds ratio; CI = confidence interval
